# Case Report: First longitudinal study of a patient with CALR positive clonal hematopoiesis of indeterminate potential developing into pre-fibrotic myelofibrosis

**DOI:** 10.3389/fonc.2023.1176173

**Published:** 2023-05-08

**Authors:** Lasse Kjær, Vibe Skov, Morten Kranker Larsen, Tobias Idor Boklund, Morten Andersen, Maria Kefala, Trine A. Knudsen, Christina Schjellerup Eickhardt-Dalbøge, Thomas Stiehl, Johanne Gudmand-Høyer, Jordan Snyder, Morten Holmström, Mads H. Andersen, Johnny T. Ottesen, Christina Ellervik, Hans C. Hasselbalch

**Affiliations:** ^1^ Department of Hematology, Zealand University Hospital, Roskilde, Denmark; ^2^ Centre for Mathematical Modeling - Human Health and Disease, IMFUFA, Department of Science and Environment, Roskilde University, Roskilde, Denmark; ^3^ Department of Surgical Pathology, Zealand University Hospital, Roskilde, Denmark; ^4^ Institute for Computational Biomedicine – Disease Modeling, RWTH Aachen University, Aachen, Germany; ^5^ National Center for Cancer Immune Therapy, Copenhagen University Hospital Herlev, Herlev, Denmark; ^6^ Department of Data and Data Support, Sorø, Region Zealand, Denmark

**Keywords:** case report, MPN, CALR, clonal hematopoiesis, myelofibrosis

## Abstract

Initial diagnosis of overt myeloproliferative neoplasms (MPNs) represents the juncture during clonal evolution when symptoms or complications prompt an afflicted individual to seek medical attention. In 30-40% of the MPN subgroups essential thrombocythemia (ET) and myelofibrosis (MF), somatic mutations in the calreticulin gene (*CALR*) are drivers of the disease resulting in constitutive activation of the thrombopoietin receptor (MPL). In the current study, we describe a healthy *CALR* mutated individual during a 12 year follow-up from initial identification of *CALR* clonal hematopoiesis of indeterminate potential (CHIP) to the diagnosis of pre-MF. The pre-diagnostic exponential development dynamics of the malignant clone demonstrated close correlation with the platelet counts, neutrophil-to-lymphocyte (NLR) ratio, and inversely correlated to hemoglobin and erythrocyte counts. Backward extrapolation of the growth rate indicated the potential for discovery of the malignant clone many years prior to presentation of overt disease, opening a window of opportunity for early treatment intervention. We did not find any additional mutations associated with MPNs and the current case report provides novel information regarding the development of a driver mutation and the association with blood cell counts prior to clinical manifestation of symptoms suggesting that pre-diagnostic dynamics may supplement future diagnostic criteria for early diagnosis and intervention in MPN patients.

## Introduction

The Philadelphia negative myeloproliferative neoplasms (MPNs) are clonally derived entities sub-grouped into essential thrombocythemia (ET), polycythemia vera (PV), and myelofibrosis (MF) according to their clinical features, genetic aberrations, and preferably - bone marrow morphology (1). The most prevalent somatic *Janus kinase* (*JAK2*) mutation - *JAK2V617F -* is the ‘driver’ of more than 95% of cases with PV, in addition to 50-60% of cases with ET and MF (2–4). Mutations in the *calreticulin* gene (*CALR*) are present in 80-90% of the *JAK2V617F*-negative ET and MF patients (5, 6) introducing a novel C-terminus enabling the protein to activate the thrombopoietin receptor (MPL) by homo-multimerization (7–9). The World Health Organization (WHO) has added clonal hematopoiesis (CH) to its classification system recognizing a precursor state for myeloid disease based on the entity clonal hematopoiesis of indeterminate potential (CHIP) where somatic mutations with a frequency of 2% or higher without other indications of hematological neoplasm, is identified as a pre-leukemic clone (10, 11). Clonal hematopoiesis is widely observed in the elderly and is furthermore associated with increased risk for cardiovascular disease (CVD) (12, 13). Alarmingly, we recently identified the MPN driver mutation *JAK2V617F* in 3% of the background population of which 42% resembled an early MPN phenotype even though the mutant allele burden in the vast majority was below 2% (14). Interestingly, the *CALR* type 1 and type 2 mutations appeared to be around 19 fold less frequent than *JAK2V617F* in the background population compared to the expected 5.6 fold difference estimated for MPNs. The *CALR* mutated individuals had a higher mean allele burden and accordingly 60% displayed the early MPN phenotype suggesting shorter latency for development of disease compared to the *JAK2V617F* mutation (5, 6, 14). In MPNs, clonal *CALR* mutations are considered an early event (5) and, compared to *JAK2V617F*, the clones have been suspected of a rapid growth rate as the proportion of diseased *CALR* positive individuals is higher, patients rarely present with a low mutant allele burden at the time of diagnosis, and are diagnosed at a younger age (14–17). The notion was recently further supported by a study investigating disease initiation and dynamics of clonal expansion by mathematical modeling (18). In MPNs, the dynamics of the mutant allele burden and the blood cell counts appear to be associated (19) but, to our knowledge, the association of the *CALR* dynamics with blood cell counts in the pre-MPN state has not been described by longitudinal studies so far.

The discrepancy between the prevalence of driver mutations in the background population and MPN diagnoses might involve the expected long latency from acquisition of the mutation until diagnosis (20). Increased death rate from thrombotic events during the pre-diagnostic phase because of late detection of developing MPNs would result in the disease eluding discovery suggesting a possible massive under-diagnosis of the disease (21, 22).

In this case report, we present for the first time a healthy *CALR* mutated individual with 12-year longitudinal follow-up until development of pre-fibrotic MPN, where early treatment could be initiated, demonstrating consistent exponential growth of the mutant clone independent of other somatic mutations associated with hematological neoplasms. The expansion rate of the clone suggests a narrow time window from diagnosis to full dominance of the mutated clone. Close correlation of pre-diagnostic mutant allele burden-and platelet count dynamics combined with backward extrapolation of the growth rate suggested the potential for discovery of the malignant clone many years prior to presentation of overt disease, opening a window of opportunity for early treatment intervention.

## Case presentation

In 2017, a screening study for *JAK2V617F* and *CALR* mutations was performed on samples collected in 2010 for the Danish General Suburban Population Study (GESUS) – a cross sectional cohort of the Danish background population (14). Here, a *CALR* type 1 mutation was identified in a 63-year-old male, with a mutant allele burden in peripheral blood of 0.071%.

In 1976, at the age of 29 years he had a pacemaker implantation because of intermittent bradyarrhythmias, was diagnosed with essential hypertension in 2010, and hiatus hernia in 2012. In these years, the blood cell counts were normal (**Table 1**). The patient quit smoking in 1970 at the age of 23 but information on smoking pack years was not available. From the time of identification of the *CALR* mutation, until diagnosis of pre-MF, the patient experienced no MPN related symptoms other than the elevated platelet counts, including absence of splenomegaly. Before the time of diagnosis, the patient was treated for elevated blood pressure form June 2021 with 5 mg Amlopidin daily, which was changed in January 2022 to 100 mg Losartan daily. The patient furthermore received 0.1% Mometason cream for psoriasis in February 2022 and 500 mg Metformin daily for diabetes in November 2022. At the end of November 2022, the patient was treated with 45 µg/week pegylated interferon-alfa-2a.

**Table 1:** Pre-clinical and clinical characteristics.

Date	% CALR mut	Hb g/L	Leuk (x10^9^/L)	Plt (x10^9^/L)	Eryt (x10^12^/L)	Eryt vol (vol fL)	MCV fL	LDH U/L	CRP (mg/L)	eGFR (ml/min/1,73m^2^)	Mono (x10^9^/L)	Neut (x10^9^/L)	Lymph (x10^9^/L)	NLR
20-09-2010	0.071	158	6.6	182	5.6	0.5	88	na	5.6	na	0.6	3.9	1.9	2.1
29-08-2017	1.8	166	7.9	194	5.8	0.5	87	278	5.3	80	0.56	5.29	1.91	2.8
16-04-2018	2.3	155	6.6	206	5.3	0.45	84	266	3.1	79	0.48	4.02	1.89	2.1
24-09-2018	2.6	158	6.6	220	5.5	0.49	85	280	3.4	80	0.5	4.2	1.7	2.5
19-03-2019	3	164	7.4	220	5.8	0.49	85	280	3.4	62	0.7	4.5	2	2.3
10-09-2019	3.8	156	7.2	204	5.5	0.46	84	320	4.1	59	0.6	4.3	2.2	2.0
18-11-2019	5.1	158	6.5	209	5.5	0.46	84	280	3.3	74	0.4	4.3	1.6	2.7
13-05-2020	7.1	153	6.9	251	5.2	0.44	84	290	4.9	63	0.5	4.7	1.6	2.9
14-12-2020	8.9	150	6.6	295	5.3	0.46	87	270	<2.9	77	0.5	4	1.7	2.4
24-06-2021	12	153	10.7	350	5.3	0.47	89	270	97	64	0.8	7.7	1.9	4.1
06-01-2022	15	145	7.7	355	5.0	0.45	89	300	4.7	70	0.6	5.1	1.8	2.8
29-08-2022	19	147	7.5	570	5.2	0.45	87	330	4.8	76	0.6	5.1	1.5	3.4
12-10-2022	15	147	6.4	470	5.2	0.46	88	320	4	73	0.5	4.5	1.3	3.5
01-11-2022	20	137	9.3	545	4.8	0.42	88	280	<2.9	72	0.7	6.7	1.6	4.2

Pre-clinical and clinical characteristics. Red digits indicate values above the reference range and blue indicate values below the reference range. Hb, hemoglobin; Leuk, Leukocytes; Plt, Platelets; eryt, Erythrocytes; Eryt vol, Erythrocyte volume fraction; MCV, Mean corpuscular volume; LDH, Lactate dehydrogenase; CRP, c-reactive protein; eGFR, Estimated glomerular filtration rate; Mono, Monocytes; Neut, Neutrophils; Lymph, Lymphocytes; NLR, neutrophil-to-lymphocyte ratio.

**Figure 1:**
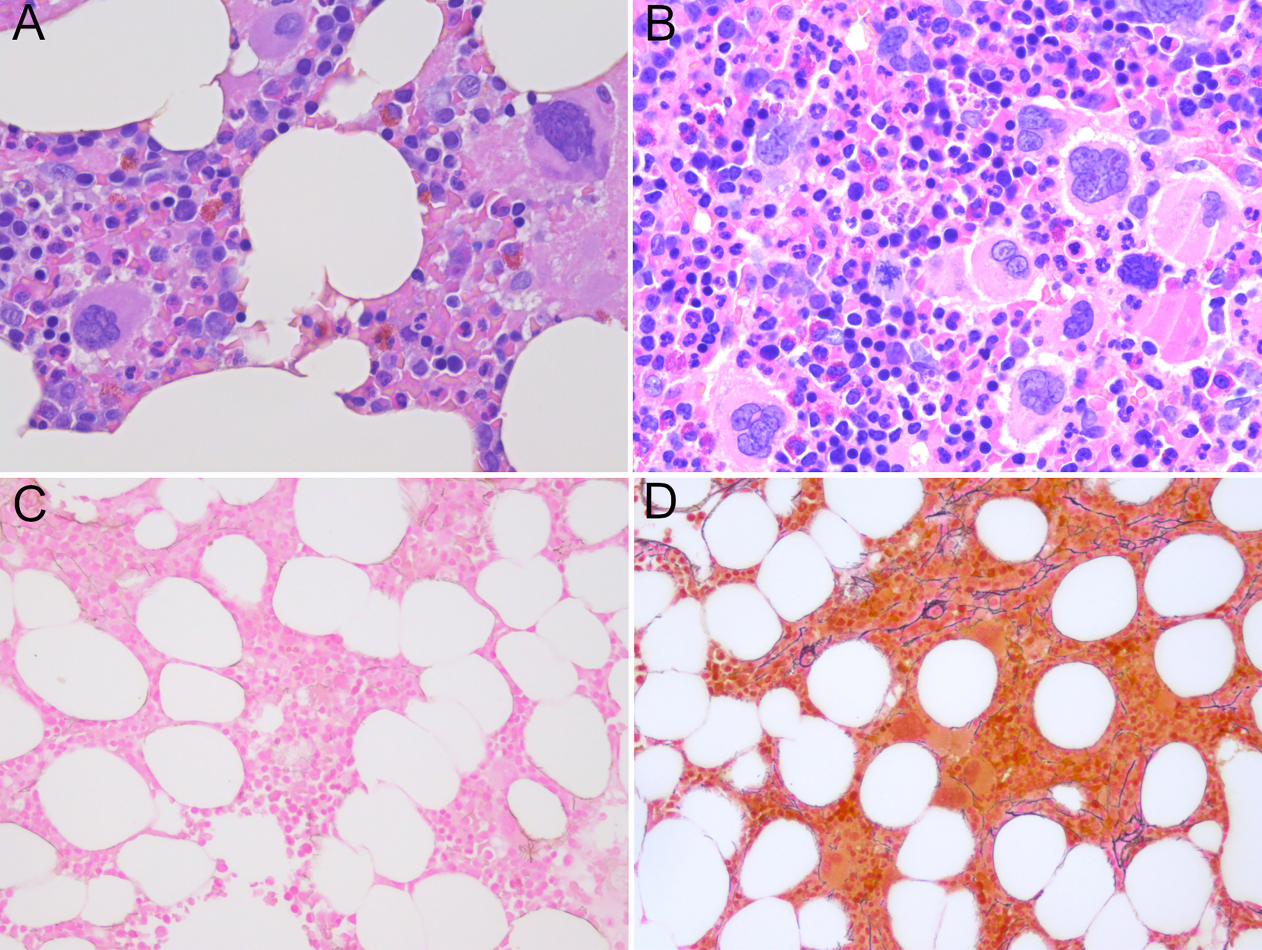
**(A)** Normocellular bone marrow not diagnostic for myeloproliferative neoplasia, only a few megakaryocytes with myeloproliferative features in sample from 2017. **(B)** Hypercellular bone marrow with clusters of atypical megakaryocytes with myeloproliferative features without fibrosis in sample from 2022. **(C)** Reticulin staining in sample from 2017 reflecting a normal bone marrow. **(D)** Loose networks of reticulin staining grade 0-1 in bone marrow sample from 2022. **(A, B)** Hematoxylin and eosin staining; **(C, D)** Reticulin staining; magnification x400).

**Figure 2:**
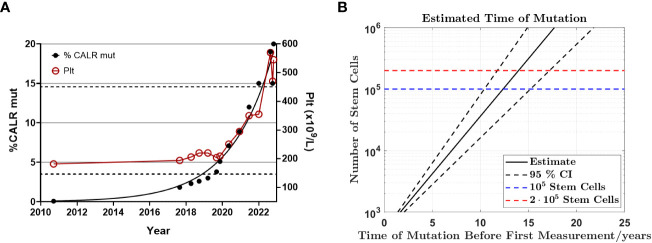
Exponential growth of *CALR* mutant allele burden from CH to MPN **(A)** Graph illustrating the mutant allele burden of *CALR* type 1 from September 2010 to November 2022 demonstrating exponential growth of the mutant allele burden and the platelet count. Note the close correlation between the *CALR* mutant allele burden and the platelet count once it exceeds 5%, including the transient drop even from 19% mutant alleles to 15% indicating a biological phenomenon rather than a technical discrepancy. The left Y-axis indicates the mutant allele burden, where black dots indicate *CALR* data points. The right Y-axis describes the number of platelets/µl and the red circles measurements of platelets. The dashed lines indicate the upper and lower boundary for the reference range of platelet count. **(B)** Backward extrapolation used to estimate when the mutation occurred. A heterozygous mutation in 1 out of 100.000 (dashed blue line) or 200.000 (dashed red line) stem cells.

**Figure 3:**
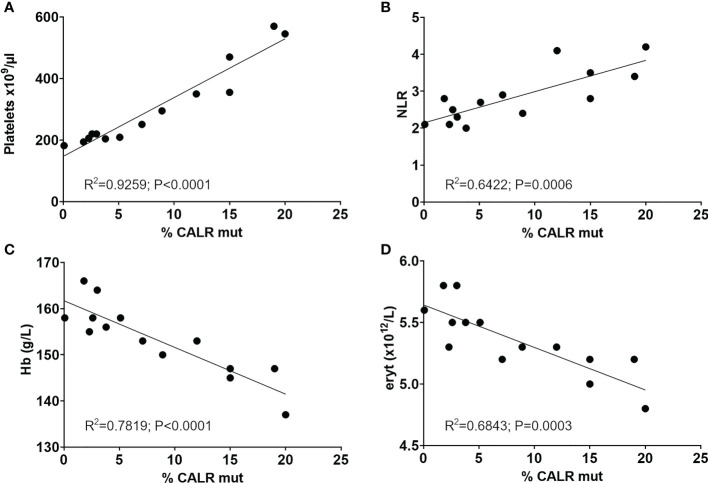
Significant correlation of longitudinal *CALR* mutant allele burden with **(A)** platelet counts, **(B)** the neutrophil-to-lymphocyte ratio, **(C)** hemoglobin and **(D)** erythrocyte count. R squared and p values from Person correlation are shown in graphs. NLR, Neutrophil-to-lymphocyte ratio; Hb, Hemoglobin; eryt, Erythrocyte count.

Subsequent to identification of the mutation in 2017 - seven years after the initial GESUS blood sample - follow-up samples and a bone marrow biopsy analysis were performed on the patient who was 70 years old at the time. The blood cell counts and bone marrow biopsy were normal although, when reviewed, early MPN features could be discerned (**Figures 1A, C**). Plasma lactate dehydrogenase (LDH) was slightly elevated, and qPCR determined that the *CALR* allele burden had increased to 1.8% (19). Thereafter, the patient was followed twice yearly with routine blood tests and a *CALR* mutation analysis. In 2018, the *CALR* mutant allele burden had increased to 2.3% and elevated plasma LDH remained the only abnormal blood parameter (**Table 1**), but at this point, the exponential growth of the mutated clone became evident (**Figure 2A**). The normal blood parameters remained within the reference range until October 2019. The Leukocyte count was briefly elevated in 2021 and at the end of 2022. In August 2022, the platelet count had increased to the diagnostic criteria for MPN and the mutant allele burden was 15-19%. A bone marrow biopsy was performed in November showing hyperplastic granulopoiesis and hyperplastic megakaryopoiesis with dense and loose small clusters of megakaryocytes demonstrating clear myeloproliferative features. This included forms with hyper-lobulated, as well as bulbous and hyperchromatic, nuclei (**Figure 1B**). In addition, silver staining revealed focally lightly accentuated reticulin fibrosis grade 0-1 reminiscent of an early pre-fibrotic myelofibrosis (**Figure 1D**). Although within the reference range, the platelet count clearly increased alongside the mutant allele burden demonstrating significant correlation (Pearson: R^2^ = 0.9259, P<0.0001) (**Figures 2A**, **3A**). Interestingly, the platelet count dynamics resembled the mutant allele burden dynamics several years prior to diagnosis with an initial increase in platelet count around 2% mutated alleles that reached a plateau phase and subsequently decreased, suggesting an intrinsic regulation of the platelet number. However, this appeared to be overwhelmed when the mutant allele burden reached 5% and platelets increased, exceeding the reference range when the mutant allele burden was above 15% (**Figure 2A**). Fitting the exponential expression $y=A\cdot e^{ax}$ , where x is the time measured in years after the first measurement, to the *CALR* allele burden (using the fit function in MATLAB’s curve fitting toolbox, version R2021b) results in A=0.103% (95% CI (0.006922%, 0.199%) ) and, $a=0.4311\;{\text{years}}^{-1}$ (95% CI $\left(0.3503\;{\text{years}}^{-1},\;0.5119\;{\text{years}}^{-1}\right)$ , with $R^{2}=0.9603$ , corresponding to a doubling time of 1.61 years (95% CI (1.35 years, 1.98 years) ) and a yearly growth rate of 53.9% (95% CI (41.9%, 66.8%) ). Assuming the assay for determination of mutant allele burden is performed in duplicate with 150 ng DNA (2 x 150 ng DNA containing 100.000 copies of *CALR)*, and that the assay has the ability to detect 3 mutated copies, the detection limit will correspond to approximately 0.003% mutated alleles. To determine the ‘window of opportunity’ for detection of the mutation, we assume that the exponential fit also describes the growth of the mutated clone prior to the first measurement, and we extrapolate the exponential fit backwards in time to the detection limit of the assay (0.003%) (14). Solving the equation y=0.003% , we find x=−8.20 years , i.e. that the mutation was detectable 8.20 years (95% CI (6.91 years, 10.1 years) based on the 95% CI of a ) before the first measurement in 2010, thus enabling detection 20.1 years (95% CI (18.8 years, 22.0 years) prior to the MPN diagnosis in August 2022. In addition, assuming that the allele burden in the peripheral blood equals the allele burden in the stem cell compartment (or at least that the time delay between the mutation happening at stem cell level and subsequently appearing in the peripheral blood is negligible on a time scale of years), and neglecting stochasticity, stem cell-specific regulations, and anti-cancer immunity, backward idealized extrapolation can be used to estimate the time of the initial mutation. If we assume that the number of stem cells is N and that the mutation is heterozygous as it normally is for *CALR* mutations, then the estimated time of mutation from extrapolation can be found by solving the equation $\text{y}=\frac{1}{2N}\cdot 100\%$ , where the factor of 100% converts the fraction of cells to an allele burden measured as a percentage, and the factor of $\frac{1}{2}$ is due to the heterozygosity. Different sources have different estimates for the number of hematopoietic stem cells in a human, and therefore we have calculated the estimated time of mutation for a wide range of the assumed number of stem cells (**Figure 2B**). A heterozygous mutation in one out of *N* = $10^{5}$ stem cells (23) (corresponding to an allele burden of 0.0005% ) is estimated to have occurred 12.4 years (95% CI (10.4 years, 15.2 years) based on the 95% CI of a ) before the first measurement, and a heterozygous mutation in one out of *N* = $2\cdot 10^{5}$ stem cells (24) (corresponding to an allele burden of 0.00025% ) is estimated to have occurred 14.0 years (95% CI (11.8 years, 17.2 years) based on the 95% CI of a ) before the first measurement, i.e. somewhere between the age of 45 and 53 for the patient based on the assumed number of stem cells (**Figure 2B**).

The mutant allele burden demonstrated a significant correlation with the neutrophil-to-lymphocyte ratio (NLR) (Pearson: R^2^ = 0.6422, P=0.0006) (**Figure 3B**). An inverse correlation was observed between the mutant allele burden and both hemoglobin (Hb) (Pearson: R^2^ = 0.7819, P<0.0001) and erythrocytes (Pearson: R^2^ = 0.6843, P=0.0003) (**Figures 3C, D**). There was no correlation between mutant allele burden and monocytes, leucocytes, plasma LDH, or renal function as assessed by eGFR (data not shown). Chromosome analysis revealed loss of the Y-chromosome in single metaphases as the only cytogenetic aberration. No additional mutations were detected in the last available peripheral blood sample by next generation sequencing (NGS) analysis of peripheral blood using the custom panel of 42 genes implicated in myeloid malignancies described by Grauslund et al. (25, 26).

## Discussion

To our knowledge, this is the first observation of a *CALR* mutation detected at a very low mutant allele burden in an individual longitudinally monitored over a 12-year period prior to developing pre-fibrotic myelofibrosis. Absence of known co-mutations involved in myeloid malignancies suggested that *CALR* was the only genetic cause of the expansion.

Recently, a study investigated the longitudinal dynamics of clonal hematopoiesis suggesting that the large majority of clones expanded at a stable exponential rate with average annual growth rates ranging from 5% to 50% per year (24). The *CALR* growth rate of approximately 54%, in the current study, exceeds the rate of expansion for the fastest clones such as *SRSF2^P95H^* and mutant *U2AF1* (24). Initial longitudinal observations of slow exponential growth for *JAK2V617F* mutated clones suggested complex growth dynamics (27) confirmed by a wide range of clonal expansion rates increasing with additional mutations (12). The growth dynamics for *CALR* remain to be studied in larger cohorts, but at least for the patient in the current study, the rapid growth rate appears to be independent of additional mutations in genes typically associated with hematological cancers and may involve other factors such as mutated calreticulin acting as a rogue cytokine (28). The *JAK2V617F* mutation is acquired early in life (29, 30) and even *in utero* (20, 31). Considering the estimated midlife *CALR* acquisition estimated both by mathematical modeling (18) and the current study, the reported in utero origin of *CALR*, recently confirmed in a set of monozygotic twins (32), suggests that the growth dynamics for *CALR* may be as irregular as seen for *JAK2V617F* (20). Assessing clonal growth rates requires a set of assumptions. For predicting the time of mutant acquisition of *DNMT3A* variants (20), the assumption that the clones expanded at a steady rate meant that the mutations initially appeared to have occurred several years before birth. However, the underlying age dependent deceleration in growth rate for *DNMT3A* clones was not observed for clones with variants in TET2 (20) highlighting the importance of longitudinal monitoring of clone expansion. In the current study, we make necessity assumptions when neglecting stochasticity, stem cell specific regulations, and anti-cancer immunity when determining the time of mutation, but we are reasonably confident about our assumptions as for the current patient the growth rate appears constant over the entire 12-year period and thus we simplify the potentially complex growth of the clone. However, we acknowledge that this may not necessarily reflect the *CALR* development in other patients. Of note, the bone marrow biopsy appeared normal 5 years prior to the MPN diagnosis when the mutant allele burden was around 2%, although with rare MPN features. Prominent MPN features were present when the allele burden had exceeded 15% corresponding to 30% of nucleated cells as *CALR* mutated individuals rarely present with dominant homozygous clones (15). The estimated doubling time of the *CALR* clone of 1.61 years suggests that the vast majority of nucleated cells in the blood will consist of the mutated clone 1-2 years after initial clinical presentation, although differences between cell lineages are expected (33).

We have previously observed a close association between the dynamics of the blood cell counts and the mutant allele burden in *CALR* mutated MPN patients (19). In the current patient, the dynamics of the platelet count bore a striking resemblance to the mutant allele burden and indicated disease development even when parameters were still within the reference interval. Consequently, such pre-diagnostic alterations might supplement future diagnostic criteria, enabling an even earlier MPN -diagnosis prior to potential thrombotic events.

The MPNs are driving – and driven by – inflammation, reflected in the positive correlation between the *CALR* mutant allele burden and the chronic inflammation proxy: NLR, in this case study. This supports findings of increased NLR in *CALR* positive individuals compared to mutation negative and further suggests persistent correlation over several years during development of the mutated clone (34). We did not observe the previously reported negative associations with eGFR suggesting either a mild impairment, not yet reflected by eGFR, or the presence of additional factors having impact on kidney function.

Although CH is recognized as a clinical entity associated with increased overall mortality, CVD and myeloid disease, treatment in the absence of a hematologic disorder according to current guidelines is a controversial subject. Clonal hematopoiesis is not a single entity but is associated with a wide variety of mutations some clearly representing a critical precancerous state – especially MPN driver mutations in *JAK2, CALR*, and *MPL* genes require extra scrutiny (7). It is becoming increasingly evident that the MPN diagnosis only reflects the juncture during the clonal evolution where symptoms or complications prompts an afflicted individual to seek medical attention (35). Here, we detected a *CALR* mutation many years prior to clinical manifestation and were able to initiate early intervention and treatment at the very instant the criteria for the MPN diagnosis were achieved, prior to potential thrombotic – and thromboembolic complications. Recently, eradication of a double mutant-driven pre-leukemic clone prevented leukemic onset in a mouse model (36) proving proof of principle for the elimination of the malignant clone prior to overt disease and extending the concept of early intervention in MPN patients (37). Transition from ET to post-ET MF is associated with increased mutant load of *CALR* (38) providing further incentive for reducing the mutant allele burden, keeping in mind that its reduction is much more difficult and time-consuming when the mutated fraction is high, exposing the patient to unnecessary complications. It is likely that there will be a wide variety of developmental trajectories for *CALR* mutated clones depending on intrinsic and extrinsic factors where some carriers may never develop MPN. In the current case, presence of the mutation in 30% of circulating nucleated cells at clinical manifestation, and the observed growth rate of the clone, suggested a very short time-period from MPN diagnosis until eclipse of the normal cells. Considering the aggressive expansion, the early intervention may have prevented thromboembolic events, but understanding risk stratification requires further studies on how the development of *CALR* clones associate with clinical events. We need to start the discussion on how to handle the driver mutation CHIP patients with either dormant clones only requiring regular monitoring or rapid clonal expansions prompting ‘preventive’ medicine interventions such as aspirin to reduce the risk of thromboembolic events or even attempting to deplete the malignant clone by interferon treatment (19, 39). Therefore, the current case report highlights the urgent matter of re-considering early intervention and disease prevention when driver mutation and the dynamics of the blood cell counts, even within the reference interval, suggest progression.

## Data availability statement

The original contributions presented in the study are included in the article/supplementary material. Further inquiries can be directed to the corresponding author.

## Ethics statement

The study was approved by the Regional Committee on Health Research Ethics and the Danish Data Protection agency. The patients/participants provided their written informed consent to participate in this study. Written informed consent was obtained from the individual(s) for the publication of any potentially identifiable images or data included in this article.

## Author contributions

LK, VS, ML, TK, CS, MH, MA, and HH conceived and designed the study. HH and CE managed the patient. MK carried out the pathological analysis. VS carried out the NS analysis. TB, MA, TS, JG, JA and JO did the mathematical calculations. LK wrote the manuscript. All authors contributed to the article and approved the submitted version.
